# Perimortem Skeletal Sharp Force Trauma: Detection Reliability on CT Data, Demographics and Anatomical Patterns from a Forensic Dataset

**DOI:** 10.3390/biology11050666

**Published:** 2022-04-26

**Authors:** Sandra Braun, Lara Indra, Sandra Lösch, Marco Milella

**Affiliations:** Department of Physical Anthropology, Institute of Forensic Medicine, University of Bern, 3008 Bern, Switzerland; sandra.braun@irm.unibe.ch (S.B.); lara.indra@irm.unibe.ch (L.I.); marco.milella@irm.unibe.ch (M.M.)

**Keywords:** forensic anthropology, forensic sciences, sharp force trauma, skeletal lesions, postmortem computed tomography (PMCT), imaging techniques, anaglyph glasses, suicide, homicide

## Abstract

**Simple Summary:**

The increased use of computed tomography images in forensic anthropology is easily explained with a variety of benefits: among other reasons they are digitally stored, they can easily be shared and they are non-invasive. However, it is not clear how suitable these images are for forensic anthropologists to detect sharp force trauma. Therefore, we analyzed computed tomography images, by observing digital images of 41 forensic cases in different viewing modalities. In addition, we looked for anatomical injury patterns in the soft- and hard-tissues and any significant correlations between the manner of death (suicide or homicide) with different parameters. Our findings indicated a superiority of viewing the images in 2D, but not all bone lesions were detected. The manner of death was significantly correlated to some of the parameters, which could be extrapolated to future forensic anthropological cases. We promote the inclusion of imaging training into the anthropological curricula.

**Abstract:**

The increasing importance of trauma analysis by means of postmortem computed tomography (PMCT) is insufficiently reflected in forensic curricula, nor are best practice manuals available. We attempt to detect sharp force bone lesions on PMCT of closed forensic cases with the aims of assessing errors and pointing out patterns in anatomical location and manner of death (MOD). We investigated 41 closed sharp force fatality cases, with available PMCT and forensic reports. Two observers with different radiological training assessed the lesions on PMCT scans (2D and 3D) for comparison with the reports. Between 3% (suicides) and 15.3% (homicides) of sharp force injuries caused visible bone lesions. While our observations were repeatable, each forensic investigation left a similar number of bone lesions undetected. Injury patterns differed between MOD, with thoracic bone lesions being most frequent overall. Soft tissue injury location varied between the MOD. Associations between MOD and age as well as number of injuries were significant. The detection of bone lesions on PMCT for untrained forensic specialists is challenging, curricula and pertinent manuals are desirable. With the low frequency of bone lesions compared to soft tissue injuries, we should be aware when analyzing decomposed bodies.

## 1. Introduction

Traumatic injuries are among the most frequent causes of death worldwide, annually accounting for over five million fatalities, more than a quarter of which owing to self-directed and interpersonal violence [1]. It is therefore not surprising that the analysis of skeletal lesions is of paramount importance in casework, and their interpretation one of the main steps during forensic examination [2]. Trauma has been defined as an accidental or inflicted injury following a *‘harsh contact with the environment’* [3]. Among the various mechanisms of trauma, sharp force features distinctive morphological traits, which can allow us to draw information about the circumstances and the manner of death [4,5], considering the presence or absence, number and location of bone lesions, as well as the overall injury severity [6,7,8]. By definition, sharp force is inflicted with a sharp object that is edged, pointed or beveled [9], causing incisions and punctures [2], as well as chop or hack marks [9]. Focusing on sharp force trauma, which is often encountered during forensic examination [8,10], we applied an approach similar to Spies and colleagues, testing the detectability of traumatic bone lesions [11].

### Imaging Techniques in Forensic Sciences

Nowadays, perimortem skeletal lesions are often assessed radiographically, applying a variety of modalities [12]. Imaging techniques such as computed tomography (CT) were originally used for clinical diagnostic purposes [13,14,15]. The first forensic CT scans were published in 1977 to describe gunshot injuries to the head [16]. Since the early nineteen-nineties, a number of institutes have been introducing radiological data into forensic casework [17,18,19,20,21]. At the Institute of Forensic Medicine (IRM) Bern, Switzerland, the addition of imaging techniques to traditional autopsy started in the mid-nineteen-nineties [18,19,22]. Around the turn of the millennium, the VIRTOPSY^®^ project was established in Switzerland, routinely complementing autopsy with postmortem CT scans and radiological reports, in addition to three-dimensional surface scanning as well as magnetic resonance imaging (MRI) [18,23,24,25,26]. There is considerable additional insight in the combination of traditional and virtual approaches to avoid misdiagnosis [27].

Virtual bone models are advantageous for the digital storage, the easy sharing among forensic experts for repeated analysis and their admitted use in court [12,19,21]. Contrasting with clinical imaging, motion artefacts are not an issue with postmortem CT [13,15]. Moreover, in postmortem CT scanning protocols, the adverse effects of high radiation exposure are negligible, thus enabling enhanced image resolution and quality [13,15]. Owing to the high resolution, the detection of bone lesions in places not routinely dissected, foreign objects and/or gas in a body, are further advantages of postmortem CT scans [18,28]. The resulting enhanced sensitivity enables experts to detect minute kerfs on skeletal elements, especially in the case of micro-CT [29,30,31,32] and explains why the CT modality outweighs some of the other modalities in terms of resolution [11,33].

The use of radiological technologies in forensic anthropology allows non-invasive investigations of human remains with adhering soft tissue [34], where trauma lesions may not be macroscopically identifiable. In cases of burnt or charred human remains [35], as well as mummified remains [36,37,38,39], imaging techniques can support the forensic analysis and can be more suitable than the osteological analysis [35,40].

A limitation of the CT technology in forensic sciences is the depiction of soft tissues, for which the MRI modality is generally used [18]. Furthermore, the cost-effectiveness of a CT scanning device acquisition and operation, especially for institutes with relatively few forensic cases, could be a hurdle [41]. A best practice manual based on standardized curricula [11] for the use of virtual imaging of bone in forensics would be desirable [12].

Oftentimes, retrieved human remains are incomplete or fragmented [42], rendering the reconstruction of the circumstances of death challenging. This challenge holds true for both forensic and bioarcheological contexts, as the perimortem occurrence of sharp force circumscribes a wider timespan [43,44]. The impact in the two contexts, however, differs: in a forensic context, the assessment of such trauma may have legal consequences [45], while in a bioarcheological context, skeletal sharp force lesions are imminent and objective evidence of violent conflicts [46,47], even though the occurrence of the trauma remains unknown [43].

The study of postmortem CT data may help forensic anthropologists with inferring findings from fleshed corpses to cases without any or with little soft tissue. Consulting forensic cases with complete autopsy and radiology reports can be helpful to deduct the circumstances of death. Closed cases, in this instance involving sharp force trauma, may allow the deduction of circumstances in unresolved forensic cases. Hence, it is important for forensic anthropologists to test the feasibility to detect traumatic sharp force lesions [10], taking any previous experience with imaging techniques or radiological training into account. 

A previous study suggested that the detection of blunt force trauma from radiological imaging can be challenging for forensic anthropologists without any radiological training or experience [11]. It was shown that radiological training might be more beneficial than general, ‘analogous’ experience [11]. Despite the increasing use of imaging techniques in forensic anthropology [12,48], pertinent training is rarely part of the curriculum [11]. The asserted relative paucity of research focusing on the validation of forensic trauma analysis [12] may be a consequence of forensic specialists not being confident with the virtual modality. Osteological methods should be comprehensively validated on a variety of modalities to increase the level of familiarity with which forensic experts handle virtual data, and to assess the errors associated with modalities and observers.

Based on the above, this study aims to explore the reliability of CT imaging in depicting sharp force trauma, and at the same time to investigate the informative value of skeletal sharp force trauma as proxies of ante mortem traumatic events. Specifically, we address the following aims:

(a) We investigate the repeatability and feasibility in detecting skeletal sharp force lesions on CT data.
(b) We analyze the correlation of skeletal sharp force lesions with soft tissue injuries pertaining to the same traumatic event. Furthermore, we analyze associations with anatomical regions and tool dimensions in interpersonal vs. self-inflicted violence.

## 2. Materials and Methods

### 2.1. Materials

Our sample includes 41 cases presenting with soft tissue sharp force lesions and for which volume-rendered tomographic data (full-body or partial body scans) were available, as well as forensic radiology and autopsy reports. We included individuals only if the sustained trauma was fatal, in order to ascertain the sharp force had occurred perimortem. We excluded injuries related to medical interventions following trauma infliction, as well as healed fractures. Our specimens presented with cut, stab and/or chop wounds. We retrieved information on each case from the institutional IRM database. Between 2009 and 2013, tomographic scans were acquired with a Somatom Emotion 6 (Siemens, Berlin/Munich, Germany), with the scanning protocol of 130 kV, 30 to 90 mA for the head and 87 to 162 mA for the postcranium and slice thickness of 1.25 mm. Between 2013 to 2020, a Somatom Definition AS 64 (Siemens, Berlin/Munich, Germany) was used with scanning parameters of 140 kV, 118 to 216 mAs and slice thickness of 1 mm. Reconstruction parameters of the matrix of 512 × 512 pixel was a field-of-view (FoV) of between 220 and 280 mm for the head and between 400 and 780 mm for the full-body scans. We reconstructed and visualized all tomographic with PACS IDS 7 v. 20.2.8.3353 (Sectra, Linköping, Sweden).

For the purpose of this study, we anonymized all individual data and solely retained information regarding sex, age-at-death, manner of death (suicide/homicide), number of soft tissue injuries and presence and number of bone lesions, as well as affected body region(s) for soft tissue and bone lesions. The institutional agreement with the local investigative authorities enables us to present anonymized data.

**Figure 1:**
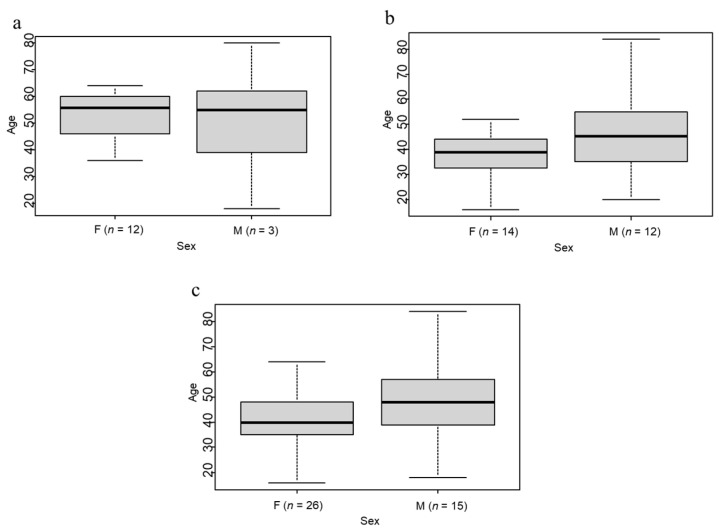
Boxplots of sex and age in the subsamples of suicide (**a**) and homicide (**b**) and the complete sample (**c**).

The resulting dataset includes 41 forensic cases (15 suicides and 26 homicides) commissioned to the IRM of the University of Bern, Switzerland between 2009 and 2020. Figure 1 shows the demographics of the sample.

We used the soft tissue injuries and bone lesions noted in the autopsy and radiology reports as the baseline. The combined reports recorded 623 soft tissue injuries and 71 bone lesions for the 41 individuals. Suicide cases (*n* = 15) totaled 198, homicide cases (*n* = 26) 425 soft tissue lesions. We list all 41 cases in Table 1, outlining the numbers of soft tissue vs. bone lesions and the average percentage of lesions per case penetrating to the bone.

### 2.2. Methods

We did not analyze the reports before engaging in the data collection, in order not to bias our observations. Data were collected by two observers (SB and LI, henceforth observer 1 and observer 2), both PhD candidates in forensic anthropology at the IRM. Observer 1 had two years of experience with virtual skeletal images while observer 2 had no previous experience apart from introductory training modules. The study design included the following steps:

#### 2.2.1. Repeatability and Feasibility

**Figure 2:**
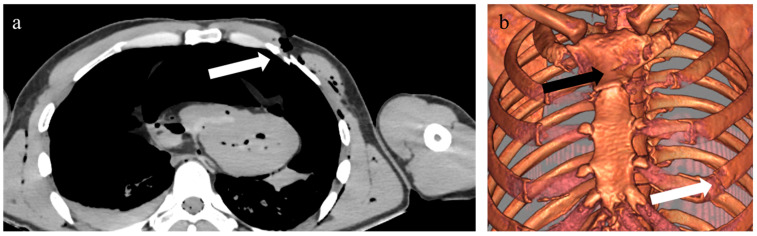
Two-dimensional (**a**) and three-dimensional (**b**) view of a sharp force homicide postmortem CT. White arrows indicate the same lesion on the left fourth rib and a sternal lesion (black arrow).

Observer 1 performed the tomographic study of all 41 cases by visualizing the tomographic data by means of two-dimensional tomographic slices (Mode1) along the transverse plane (Figure 2a) and three-dimensional reconstructions for each individual (Figure 2b). Mode2 consisted of the three-dimensional modality without anaglyph glasses, Mode3 with the aid of conventional anaglyph glasses. For each modality, observer 1 performed the data analysis twice at an interval of two weeks. For each case, we noted the presence, number and position of soft tissue and bone lesions. Observer 2 replicated the procedure on a subsample of 15 randomly selected cases, analyzing the cases once on each modality (Mode1, 2 and 3), at an interval of two weeks between modes. We then used these data to calculate the degree of error between observations (intraobserver error), observers (interobserver error) and modalities (intermodality error).

Next, we compared our observations with the information on the bone lesions contained in the autopsy and radiology reports (N = 71). The aim was to indirectly check the feasibility of such detection and, furthermore, explore the reliability of the different observation modalities in the detection of bone lesions. For an in-depth comparison of our observations with both forensic reports, we selected twelve cases with the most specific information about the exact position of bone lesions. This allowed us to explore, for this subset, the potential error between our observations and those from the autopsy and/or radiological analysis. 

#### 2.2.2. Further Analyses

Furthermore, we explored the possible differences between suicides and homicides in the presence, number and location of bone lesions in comparison to soft tissue injuries, as well as the relative exposure to injuries of different anatomical regions (head and neck, trunk, abdomen, upper extremities, lower extremities). We also attempted to compare the possible association between manner of death, blade dimensions and blade edge types (smooth or serrated), as outlined in Table 2. In 20 cases (twelve suicides and eight homicides), we found a sufficiently detailed description of the knives to analyze the dimensions of the blade. In the remaining cases, the tools were either not described in terms of dimensions or had not been retrieved on the crime scene. We included semi-sharp force into sharp force even though these terms are sometimes used separately [49]. Semi-sharp force impacts result in chop wounds caused by big-sized edged tools, e.g., axes [2,10,50]. No detailed information was available regarding size and shape of axes used to inflict semi-sharp force trauma. 

Finally, we combined a number of parameters and test for any significant associations. These parameters were: the presence and number of bone lesions; the number of soft tissue injuries; the number of affected anatomical regions; age-at-death; manner of death; sex; and tool dimensions. We assumed a statistical significance for a *p*-value ≤ 0.05.

We quantified intraobserver, interobserver and intermodality errors by means of a Cohen’s [51] weighted Kappa test [52] with a confidence level of 0.95, performed with the *DescTools* package [53] for R (v. 4.1.4). The interpretation of the *κ*-values (Cohen’s Kappa) followed Landis and Koch [54]. Accordingly, *κ*-values from 0.41 to 0.6 represent a moderate agreement, from 0.61 to 0.80 *κ*-values substantial and perfect between 0.81 and 1.00. We tested the differences between suicides and homicides in the location and number of bone lesions and associated tool class with Fisher’s and Mann–Whitney tests, performed with the R package *psych* [55]. We also applied Fisher’s and Mann–Whitney tests for eta^2^-values to explore demographic differences (sex and age-at-death, respectively) between manners of death. Moreover, we tested the association of tool dimensions to manners of death with a Kruskal–Wallis test. The visual representation of the relative density of the anatomical pattern in the presence of sharp force trauma followed Milella and colleagues [46]. 

In some of the analyses involving the number of soft tissue lesions, we presented our results with and without one case (suicide case no. 15) featuring 133 soft tissue injuries to the neck and thorax, since this exceptionally high number of injuries in a suicide could bias the results.

## 3. Results

### 3.1. Repeatability and Feasibility

The intraobserver agreements range between substantial and almost perfect agreement, while the interobserver agreements fall between moderate and perfect agreement (Table 3). The intermodality error tests between the different viewing modalities highlight a substantial agreement. 

**Figure 3:**
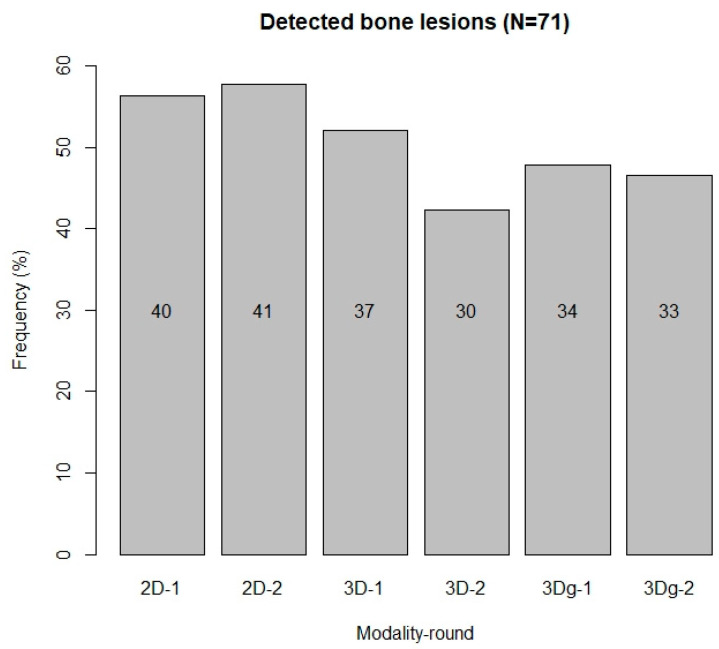
Number and frequency of detected bone lesions in all observations of all modalities (2D, 3D and 3Dg). 2D-1: first observation on the 2D modality; 3Dg-2: second observation on the 3D modality with anaglyph glasses.

Figure 3 shows the numbers and frequencies of bone lesions detected in this study, compared with those listed in the original reports. Of the 71 bone lesions in the complete sample as described in the reports, the highest number (*n* = 41, 57.8%) was detected in the second observation of Mode1 (two-dimensional slices), followed by the first observation of Mode1 (*n* = 40, 56.3%). The second observation of Mode2 (3D reconstructions without anaglyph glasses) yielded the lowest number (*n* = 30, 42.3%) of detected bone lesions.

In Table 4, we outline the twelve individuals with the most detailed injury descriptions in the autopsy and the radiology reports, for the direct comparison of the bone lesions described therein with our observations. Out of the 38 bone lesions in these twelve cases, 19 (50%) were recorded in all three investigations (autopsy and radiology reports, as well as our observations). 

All three investigations detected all bone lesions in four of the twelve cases (1, 4, 25, 31). In another four cases (20, 33, 37, 39), the radiology report mentioned bone lesions undetected during autopsy. In seven cases (2, 27, 33, 34, 37, 39, 40), bone lesions recorded during autopsy were not described in the radiology reports. Bone lesions remained undetected in six cases (2, 27, 33, 37, 39, 40) in which the autopsy and/or the radiology reports had mentioned them. Overall, the two-dimensional modality proved slightly better suited to detect the bone lesions of the twelve cases. 

While both the autopsy and the radiographic analysis did not detect one bone lesion to the head and neck region, respectively, our observations of the tomographic scans detected all bone lesions to the head and neck region among the twelve cases. We did not detect six bone lesions to the thorax, though, while in the forensic autopsy and radiology reports, three and five bone lesions on the thorax were undetected, respectively. One bone lesion to the upper extremities remained undetected during autopsy, while the radiological investigation and our observations spotted it. Table 5 summarizes the undetected bone lesions of the three different investigations from the twelve cases.

### 3.2. Further Analyses

Table 6 presents the anatomical distribution of the soft tissue injuries and bone lesions described in the original reports. The soft tissue injuries in the suicides were mostly located on the head and neck region (*n* = 109), followed by the thorax (*n* = 70), the abdomen (*n* = 15) and the upper extremities (*n* = 4). Considering the available data, the frequency of soft tissue injuries vs. bone lesions changes according to the considered anatomical area. The highest frequency of bone lesion is at the level of the thorax and the head and neck region. The opposite pattern characterizes the abdomen and the lower extremities. 

When we excluded suicide case no. 15 with 133 soft tissue injuries, the total injuries among the suicides amounted to 65; head and neck region (*n* = 29), thorax (*n* = 17), abdomen (*n* = 15) and upper extremities (*n* = 4). Among the homicides, the highest number of soft tissue injuries affected the thorax (*n* = 162), followed by the upper extremities (*n* = 110) and the head and neck region (*n* = 108). Injuries to the abdomen and lower extremities were scarcer (*n* = 27 and *n* = 18, respectively).

**Figure 4:**
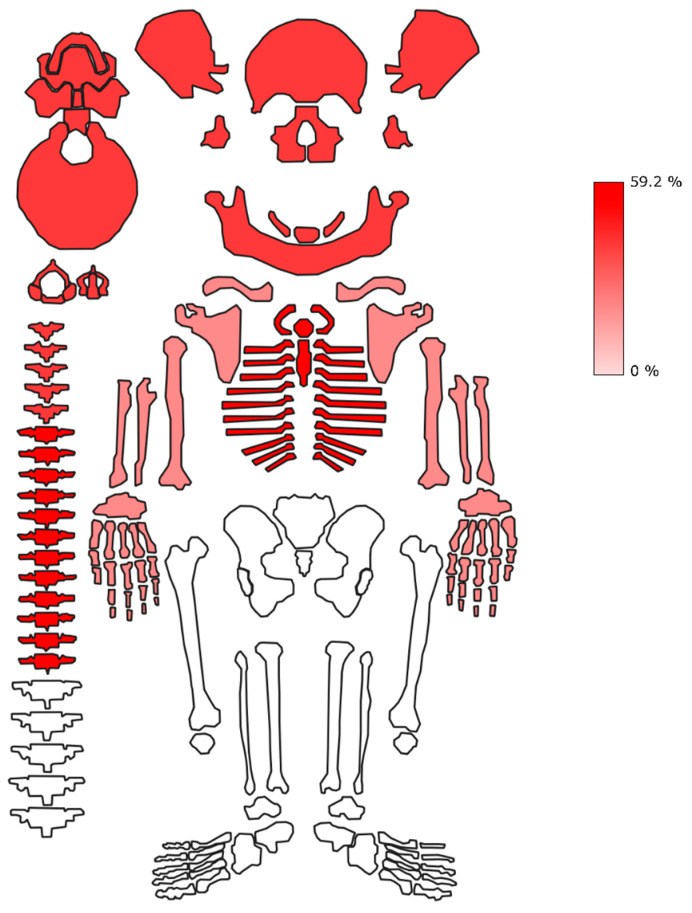
Anatomical location of bone lesions in the complete sample, expressed in frequencies (%) of total number of reported trauma (N = 71). Head and neck and thorax show the highest density of skeletal exposure. Conversely, no bone lesions are present in the abdomen and the lower extremities.

Contrasting with the soft tissue lesions, the frequency of sharp force injuries penetrating to the bone across the sample was lower in suicides than in homicides (3.0% vs. 15.3%). Excluding suicide case no. 15, the frequency of bone lesions across the sample was 9.2%. The majority of the complete sample’s bone lesions was located on the trunk (59.2%), followed by the head and neck (38.0%) and the upper extremities (2.8%), as shown in Figure 4. All bone lesions in the suicides were located on the thorax and among the homicide cases, the highest frequency of bone lesions was also found on the thorax (55.4%), followed by 41.5% on the head and neck region and 3.1% on the upper extremities. No suicidal bone lesions were located on the head and neck region, the abdomen and the extremities. Similarly, no bone lesions were present on the abdomen and lower extremities in homicide cases.

Apart from the razor blade and the scalpel used for one case of suicide each, the tools used to cause the injuries included a variety of knives (kitchen, pocket, hunting, butcher’s, bread, flick and carving knives), with serrated or smooth blades. The length and width of the blades ranged between 7 and 30 cm and between 1 and 4.5 cm, respectively. The subset of 20 cases usable for the analysis of the tool dimensions amounted to a combined number of 221 soft tissue injuries (suicides: *n* = 181; homicides: *n* = 40), twelve of which penetrated to the bone (suicides: *n* = 4; homicides: *n* = 8). The majority of the 221 injuries (*n* = 179, 81.0%) was caused with a category 2 tool. We found a difference between the tool dimensions in suicidal and homicidal soft tissue injuries: of the 181 suicidal injuries, 157 (86.7%) were inflicted with category 2 tools, while only 22 of the 40 homicidal soft tissue injuries (55%) were caused by a category 2 tool. Another ten homicidal injuries (25%) were inflicted with a category 4 tool and the remaining eight injuries (20%) with a category 3 tool. In contrast to the tools causing soft tissue injuries, those causing bone lesions were mainly category 4 tools. All suicidal and the majority of homicidal bone lesions (*n* = 6, 75%) were caused with category 4 tools, the remaining two homicidal bone lesions with a category 2 tool. We outline the results of the tool dimension analysis in Table 7.

The use of category 4 tools for suicidal bone lesions was statistically significant (*p*-value = 0.022). The respective homicidal bone lesions, in contrast, including category 2 and 4 tools, yielded no significant result (*p*-value = 0.448). Both manners of death combined were not significant, with a *p*-value of 0.190. 

**Figure 5:**
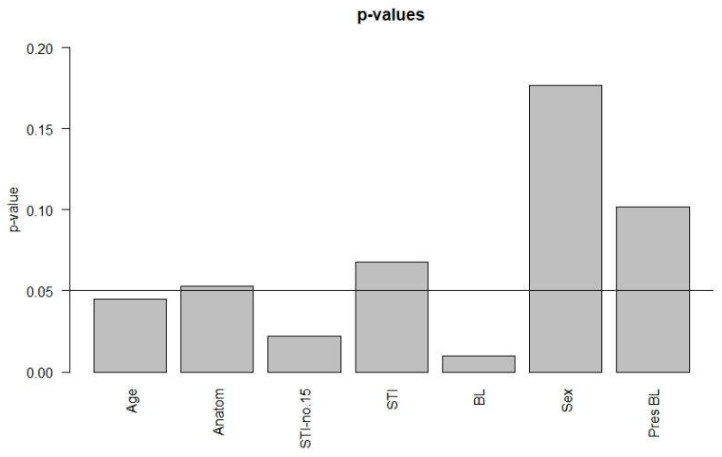
*p*-values of associations. Significant: age-at-death (age), the number of soft tissue injuries excluding case no. 15 (STI-no.15) and the number of bone lesions (BL).

Moreover, we investigated the association of various parameters (manner of death, age-at-death, number of affected anatomical regions, number of soft tissue injuries, presence and number of bone lesions and sex) for significance. We obtained significant *p*-values for the association of the manner of death with age-at-death, with the number of soft tissue injuries (excluding case no. 15) and with the number of bone lesions. In contrast, the association between the manner of death with the number of affected anatomical regions and the number of soft tissue injuries including suicide case no. 15, respectively, did not yield significant results. The eta (*η*)^2^-values between 0.681 and 0.696 provide strong effect size for all associations. For the associations between the manner of death and sex as well as the presence of bone lesions, respectively, the *p*-values were not significant. We summarize the *η*^2^- and the *p*-values in Table 8 and Table 9 and present the *p*-values graphically in Figure 5.

## 4. Discussion

The increasing availability of imaging data and their use by forensic anthropologists [12,39,56,57] result in a cautionary note regarding the usability of this data type for trauma research if not coupled with adequately trained observers. We present a point-by-point discussion of the results from the different analyses.

### 4.1. Repeatability and Feasibility

While Mode3 (3D reconstructions with anaglyph glasses) was the best viewing modality within observer 1, the agreement between the two observers on that modality was only moderate. This could point to personal aptitude to use anaglyph glasses, based on individual eyesight. Observer 1 as a habitual spectacle wearer could have handled anaglyph glasses differently from observer 2, who does not habitually wear glasses. In fact, observer 2 reported some degree of dizziness after the observations while wearing anaglyph glasses. This could explain the relatively low degree of agreement between both observers in this mode, especially since Mode1 (2D slices) and Mode2 (3D reconstructions without anaglyph glasses) resulted in perfect agreement between them. Before introducing anaglyph glasses into forensic casework, future observers could thus be advised to test their personal affinity for this modality, considering their eyesight. 

The generally superior performance of Mode1 in the direct comparison of detected bone lesions with the forensic reports is surprising. We expected a superiority of the 3D reconstructions with the ability to rotate and zoom. However, minute bone lesions were more obvious on the 2D slices, while on the 3D reconstructions, due to contrast issues, we faced greater challenges spotting them. Furthermore, on the 2D slices, the observer’s view is limited to the expanse of the present slice, while on the 3D reconstructions, the observer needs a clear focus on bone lesions. Hence, while Mode1 and Mode2 performed best between observers and Mode3 was superior in the agreement within observer 1, Mode1 was superior in the direct comparison with the forensic reports.

Regarding the comparison with the combined forensic reports, observer 1 did not detect between 30 and 38 of the 71 bone lesions (42.2% to 53.5%), depending on the modality. These findings demonstrate the difficulty to interpret skeletal lesions from tomographic data, even in the presence of sound anatomical knowledge and previous experience with radiographic data. This difficulty could partly be due to the specific challenge with the brittle and usually fragmented ribs in forensic casework, depriving forensic anthropologists of experience with intact thoracic bones. Although to our knowledge, no other studies analyzed the detectability of bone lesions in sharp force trauma on CT data, our outcomes are comparable to those from Spies and colleagues [11]. This recent experimental study analyzed blunt force fractures in piglets on different imaging modalities and compared the observations to the macerated remains of the piglets, assuming the latter as the standard. Although results obtained from experimental vs. cases from non-controlled environment studies may differ considerably [58], we concur that the detection of fractures on virtual images can be challenging for forensic anthropologists. The authors [11] found that the difficulties of trauma detection on tomographic data decreased with radiological training and experience. This highlights the need for specific training in radiological analysis to strengthen the skills of experts in this field [11].

While the above reported results on the detectability of bone lesions for forensic anthropologists are sobering, we need to relativize the discrepancy between our observations and the forensic reports. The in-depth analysis of the twelve most detailed cases showed that the differences between the three investigations (autopsy, radiology and our observations) was not divergent, with each investigation leaving five to six bone lesions undetected. The discrepancy between the reports and our observations appears greater since we are using the autopsy and the radiology reports combined as a baseline. The error is less obvious if we compare our observations to the autopsy or the radiology report individually. This stresses the benefit of multimodal investigations in forensics. Nonetheless, as pointed out in previous work [11,12], our findings highlight the importance of radiological training for the ability to detect perimortem trauma on postmortem tomographic data.

### 4.2. Further Analyses

Analyzing the anatomical distribution of soft tissue injuries and bone lesions as well as other parameters relating to the manner of death can help distinguish between suicide and homicide [8]. It is worthwhile to relate the location of soft tissue injuries to bone lesions, as a general pattern could emerge. 

The observed frequency of soft tissue injuries versus bone lesions is interesting since it can be cautiously used as an approximation of the relative informativity of different anatomical regions in documenting traumatic events. Our data strongly confirm the intuitive relationship between the amount of soft tissue layers and presence of bone lesions. This is clearly demonstrated by the absence of bone lesions in the abdominal area and, by contrast, by the high frequency of bone lesions at the level of the head and neck region and the thorax. Although not surprising, this pattern is of a general interest, being relevant in the context of both forensic examinations and osteoarchaeological analyses. The total frequency of skeletally unobservable traumatic events (ca. 89%) is quite impressive and calls for caution when trying to base forensic and paleopathological reconstructions based solely on skeletal data. Clearly, one needs to consider the likely bias on these data played by the variable typology of used sharp objects, as well as in the dynamic of applied trauma (e.g., force, direction). Moreover, our sample is relatively small, features a biased sex ratio and contains individuals from a first world context. These are the unavoidable limitations of any forensic dataset, but should be carefully considered using the results of this study while discussing findings from different cultural, social and chronological settings.

The anatomical distribution of soft tissue injuries in our sample corresponds to the findings from a comprehensive review article [5], with one difference in the most common location of injuries. The greatest number of soft tissue lesions in our suicide subsample (including and excluding case no. 15) was to the head and neck, which is slightly different from the thorax as the most frequently affected body part reported by De Giorgio and colleagues [5]. In the homicide subsample, the thorax was the most frequent location for soft tissue injuries caused by sharp force, corresponding to the reports in De Giorgio and colleagues [5]. The number of soft tissue injuries in our sample (excluding suicide case no. 15) was significantly higher in homicides as opposed to suicides.

A third of the suicide cases in our sample presented with a single soft tissue injury, the average number of injuries per suicide was 4.6 ± 4.3, excluding case no. 15. This corroborates the findings in literature, reporting a frequency of a third of the suicide cases with a single injury [4] and an average of 2.4 injuries per case [5]. The average of 4.6 in our sample would increase to 13.2 ± 32.3 if we added suicide case no. 15. We found only 19.2% of homicides with a single soft tissue injury, while literature reports about 40% [4]. The average number of homicidal soft tissue injuries was 16.3 ± 17.6, while literature mentions an average of 3.8 injuries [5]. Our homicide sample thus features a greater number of cases with multiple injuries resulting in the higher mean value compared to literature. While we need to consider the very large dataset of De Giorgio et al. [5], the sample size of 65 cases in the study by Karger and colleagues [4] is similar to ours. Hence, sample size alone does not explain the considerable differences in injury frequency; we may simply face a series of homicidal overkill cases in our sample.

Regarding the 71 bone lesions in our sample, we obtained statistical significance for the number of bone lesions per manner of death, corroborating the results of Banasr et al. [6]. The thorax was the most commonly affected anatomical region for bone lesions in suicides as well as homicides in our study. This differs slightly from another study [8], which describes the head and neck region as the most common site for homicidal sharp force trauma, followed by the thorax, but it corroborates the findings of Banasr and colleagues [6]. The number of anatomical regions affected by bone lesions did not differ significantly between the manners of death in our sample. However, we consider the number of affected anatomical regions a relevant parameter for future forensic casework, even if statistical significance was not obtained [59].

The suicidal bone lesions in our sample to the sternum (*n* = 2) and the ribs (*n* = 4) corroborate the findings reported in previous work [60]. Although bone lesions in sharp force suicides are rarer than in homicides, they are not exceptional [61]. Rare cases of aggressive sharp force suicides have been reported in literature and usually point to serious mental disorders [62,63,64]. Such cases can complicate the distinction between suicide and homicide [63], making the careful analysis of the circumstances of death and of the crime scene even more important [64]. In this context, we need to mention suicide case no. 15 in our sample, presenting with 133 superficial stab wounds to the neck as well as the thorax. Although none of the injuries penetrated to the bone, it is an example of an unusual suicide.

In several analyses of sharp force suicide and homicide cases [7,65], bone and cartilage lesions were recorded in 50% and 56%, respectively, of all cases. These frequencies are similar to 58.5% in our sample. Brunel et al. [7] as well as Teifel and Rothschild [65] reported bone and/or cartilage lesions in 14.6% and 29.8%, respectively, of sharp force suicide cases, which stands in stark contrast to 40% in our study. This high frequency could indicate a greater degree of self-directed aggression in our sample. In the 70 homicide cases, the authors [7] reported bone or cartilage lesions in 52 cases (74.3%), which is slightly higher than 69.2% in our sample. Other studies from Frankfurt (Germany) [8] and from Paris (France) [6] found 66.3% and 53%, respectively, of sharp force homicides with bone lesions. 

The inclusion of cartilaginous lesions could account for the slightly higher percentage in the study by Brunel et al. [7]. Due to the general absence of cartilage in skeletonized human remains, we did not consider cartilaginous lesions (for instance between ribs and sternum) by default. Unfortunately, the study [7] does not discriminate between bone and cartilage lesions in terms of the anatomical location and quantity, thus limiting a direct comparison with our results. However, we can relate to their finding that bone lesions are a predictor for homicide [7]. Even if the presence or absence of bone lesions was not significant for the manner of death in our study, the calculated odds ratio indicates a 3.269 times higher chance of presenting with bone lesions in a homicide. Moreover, our number of bone lesions per manner of death did yield a significant result. This could imply that a perpetrator intending to stab a person ignores the location of bones and thus more likely generates bone lesions than a person committing suicide, who might avoid skeletal elements [7]. In our study, all suicidal bone lesions were caused with a category 4bα tool (blade length >15 cm, blade width >2 cm, smooth edge). The tools causing homicidal bone lesions, in contrast, included categories 2 and 4, with unspecified or serrated edges. No smooth-edged tools were described for the homicide tools. Although the choice of sharp force tools in our sample might have been coincidental, we point to the difference in pressure needed to penetrate skin with varying blades. Smooth knife blades slice through the skin at a pressure of at least 1900 g, while a serrated blade takes considerably less pressure (700 g) to penetrate skin, due to the higher energy density and the relative ease with which a serrated blade tears into skin [66].

Apart from injury patterns and tool dimensions, we analyzed age-at-death as another parameter to discern possible differences between suicide and homicide. Mean age-at-death in sharp force suicides is often higher than in homicides [5]. Indeed, we found a significantly higher age-at-death in suicides than in homicides. Sex, in contrast, was not a significant predictor of the manner of death. However, we found an odds ratio indicating a 0.3 higher time to be male in a suicide case, even if the result was not significant. This corresponds to the findings of a study from Japan, noting that 75.4% of sharp force suicide victims were males. 

Potential limitations of our study relate to the relatively small size of the analyzed sample and the confined geographical origin of the specimens. The completeness of our sample, however, allows some cautious statements, even though we do not wish to venture into other forensic fields. Forensic cases may present with a combination of different trauma, which, however, was not the focus of the present study.

## 5. Conclusions

The detection of bone lesions on postmortem CTs is challenging for forensic anthropologists with limited training and/or experience in virtual modalities, even if the observation process is repeatable. While the 2D and the 3D modalities performed best between observers, the 3D modality with anaglyph glasses was superior to the other modalities in the agreement within one observer. Surprisingly, the 2D modality was best suited in the direct comparison with the forensic reports, even though our expectations were superiority of the plastic 3D modality. A training course for anthropology students would be desirable, familiarizing them with the basic settings, possibilities and limitations of detecting skeletal anomalies on CT images [11].

Victims of sharp force homicide stand a higher chance of presenting with a bone lesion than sharp force suicide victims and are more likely to be female. Sharp force homicides present with a significantly higher number of soft tissue injuries and bone lesions and, contrasting to sharp force suicide, affect significantly younger individuals.

Across the sample and in both manners of death, sharp force bone lesions were located mainly on the thorax. Soft tissue injuries in suicides, in contrast, were located most frequently on the head and neck region, however, without leaving kerfs on the bone. The upper extremities showed very few bone lesions in relation to the soft tissue injuries. The lower extremities presented with few soft tissue injuries, none of which penetrated to the bone.

To the best of our knowledge, this is the first study investigating the detection of suicidal and homicidal sharp force lesions on CT data, analyzing the feasibility for forensic anthropologists in this task.

## Figures and Tables

**Table 1:** List of 41 cases, number of soft tissue (STL) and bone lesions (BL), with frequency (%) of lesions penetrating to the bone, age-at-death (age), sex and the manner of death (MoD).

Case No.	STL	BL	%	Age	Sex	MoD
1	1	1	100	58	m	suicide
2	1	1	100	64	f	suicide
3	1	1	100	24	m	suicide
4	1	1	100	57	m	suicide
5	1	0	0	56	m	suicide
6	2	0	0	18	m	suicide
7	2	0	0	50	m	suicide
8	4	0	0	68	m	suicide
9	4	0	0	36	f	suicide
10	5	0	0	66	m	suicide
11	6	1	16.7	39	m	suicide
12	11	0	0	39	m	suicide
13	13	1	7.7	54	m	suicide
14	13	0	0	80	m	suicide
15	133	0	0	56	f	suicide
16	1	0	0	35	m	homicide
17	1	0	0	55	m	homicide
18	1	0	0	45	m	homicide
19	1	1	100	45	f	homicide
20	1	1	100	16	f	homicide
21	2	1	50	30	f	homicide
22	2	0	0	20	m	homicide
23	3	0	0	46	m	homicide
24	4	4	100	40	f	homicide
25	6	2	33.3	34	f	homicide
26	7	0	0	23	m	homicide
27	7	5	71.4	38	f	homicide
28	8	1	12.5	32	m	homicide
29	15	1	6.7	61	m	homicide
30	9	0	0	52	f	homicide
31	17	1	5.9	38	f	homicide
32	17	3	17.6	84	m	homicide
33	19	2	10.5	42	f	homicide
34	22	15	68.2	31	f	homicide
35	24	0	0	46	m	homicide
36	27	1	3.7	43	f	homicide
37	28	5	17.9	41	m	homicide
38	34	7	20.6	55	m	homicide
39	44	3	6.8	43	m	homicide
40	60	2	3.3	51	f	homicide
41	65	10	15.4	53	m	homicide
	Total 623	Total 71	Av 26.1	Av 45.5		

**Table 2:** Tool blade categories used in this study.

Blade Length	Category	Blade Width	Category	Blade Edge	Category
Small blades *	1	1–2 cm	a	Smooth	α
7–9.9 cm	2	>2 cm	b	Serrated	β
10–15 cm	3				
>15 cm	4				

* Includes a scalpel and a razor blade.

**Table 3:** *κ*-values for the intraobserver (intraobs.), interobserver (interobs.) and the intermodality (intermod.) agreements; interpretation of *κ*-values [54].

Intraobs. [41]			Interobs. [15]			Intermod. [41]		
Obs. 1			Obs. 1 and 2			Obs. 1		
Modality	*κ*-value	Interpret.	Modality	*κ*-value	Interpret.	Modality	*κ*-value	Interpret.
Mode1	0.675	Substantial	Mode1	1	Perfect	Mode1/2	0.688	Substantial
Mode2	0.751	Substantial	Mode2	1	Perfect	Mode1/3	0.643	Substantial
Mode3	0.9	Almost perfect	Mode3	0.474	Moderate	Mode2/3	0.751	Substantial

**Table 4:** Detected (det.) and undetected (undet.) bone lesions in the autopsy and radiology reports and our observations; modality/ies with best outcome.

Case	Autopsy		Radiology		Observations		
	Det.	Undet.	Det.	Undet.	Det.	Undet.	Modality/ies
1	Sternum	-	Sternum	-	Sternum	-	All
2	Parasternal rib 6 le	-	-	-	-	Parasternal rib 6 le	All
4	Parasternal rib 5 le	-	Parasternal rib 5 le	-	Parasternal rib 5 le	-	All
20	-	C3-4	C3-4	-	C3-4	-	2D, 3Dg
25	Occipital, C1-3	-	Occipital, C1-3	-	Occipital, C1-3	-	All
27	Rib 4 ri, ribs 3-5, 7 le	-	Rib 4 ri, rib 7 le	Ribs 3-5 le	Rib 4 ri, rib 7 le	Ribs 3-5 le	3D
31	C4	-	C4	-	C4	-	2D, 3D
33	Cranium, sternum	Rib 6 le, rib 12 ri	Cranium, rib 6 le, rib 12 ri	Sternum	Cranium, sternum	Rib 6 le, rib 12 ri	2D, 3D
34	Cranium, C1-3, maxilla, mandible, zygoma ri, nasalia	-	Cranium, zygoma ri, nasalia, maxilla, mandible, C2-3	C1	Cranium, zygoma ri, maxilla, mandible, nasalia, C1-3	-	All
37	Ribs 2, 4 le, sternum, ulna ri	Humerus head le, rib 2 ri	Rib 4 le, sternum, rib 2 ri, humerus head le, ulna ri	Rib 2 le	Sternum, ribs 2, 4 le, ulna ri, humerus head le	Rib 2 ri	2D
39	Ribs 6-8 le	Rib 5 le	Ribs 5-6 le	Ribs 7-8 le	Ribs 5-6 le	Ribs 7-8 le	All
40	Parasternal rib 2 le, rib 9 le, rib 10 ri	-	Parasternal rib 2 le, rib 10 ri	Rib 9 le	Rib 9 le, rib 10 ri	Parasternal rib 2 le	2D, 3Dg

Le: left; ri: right; C: cervical vertebra/e.

**Table 5:** Number of undetected bone lesions (BL) in the three different investigations from twelve cases.

Anatomical Region	Autopsy	Radiology	Anthropology
Head and neck	1	1	0
Thorax	3	5	6
Upper extremities	1	0	0

**Table 6:** Distribution of soft tissue injuries (STI) vs. bone lesions (BL) in the complete sample, the suicide (incl./excl. case no. 15) and the homicide subgroups.

Anatomical Region	Complete Sample (N = 41)			Suicides (*n* = 15)			Excl. No. 15			Homicides (*n* = 26)		
	STI	BL	%	STI	BL	%	STI	BL	%	STI	BL	%
Head & neck	217	27	38	109	0	0	29	0	0	108	27	41.5
Trunk (Thorax)	232	42	59.2	70	6	100	17	6	100	162	36	55.4
Abdomen	42	0	0	15	0	0	15	0	0	27	0	0
Upper extremities	114	2	2.8	4	0	0	4	0	0	110	2	3.1
Lower extremities	18	0	0	0	0	0	0	0	0	18	0	0
Total	623	71	11.4	198	6	3	65	6	9.2	425	65	15.3

**Table 7:** Tool dimension categories (cat.) in number of soft tissue injuries (STI) and bone lesions (BL) in among the manners of death (MoD).

MoD	Injury	Cat. 1	Cat. 2				Cat. 3		Cat. 4			Total
		1α	2a	2aα	2aβ	2b	3aα	3bα	4b	4bα	4bβ	
Suicides (*n* = 12)	STI	5	133	24	0	0	0	6	4	5	4	181
	BL	0	0	0	0	0	0	0	0	4	0	4
	Frequency	0%	0%	0%	0%	0%	0%	0%	0%	80%	0%	2.2%
Homicides (*n* = 8)	STI	0	3	0	10	9	8	0	7	2	1	40
	BL	0	0	0	1	1	0	0	5	0	1	8
	Frequency	0%	0%	0%	10%	11.1%	0%	0%	71.4%	0%	100%	20%
Both (*n* = 20)	STI	5	136	24	10	9	8	6	11	7	5	221
	BL	0	0	0	1	1	0	0	5	4	1	12
	Frequency	0%	0%	0%	10%	11.1%	0%	0%	45.5%	57.1%	20%	5.4%

1/2/3/4: blade length; a/b: blade width; α: smooth blade; β: serrated blade.

**Table 8:** Eta (*η)*^2^- and *p*-values of Mann-Whitney-U tests.

Parameter	Suicide			Homicide			*η* ^2^	*p*-Value
	*n*	Mean	SD	*n*	Mean	SD		
Age	15	51	16	26	42.3	14	0.69	**0.045**
Anatom	6	0.4	0.5	20	0.8	0.6	0.69	0.053
STI-no.15	65	4.6	4.3	425	16.3	18	0.68	**0.022**
STI	198	13.2	32	425	16.3	18	0.69	0.068
BL	6	0.4	0.5	65	2.5	3.6	0.7	**0.01**

Bold: statistically significant; Anatom: number of anatomical regions; STI-no.15: number of soft tissue injuries excl. case no. 15; STI: number of soft tissue injuries incl. case no. 15; BL; number of bone lesions.

**Table 9:** Odds ratios and *p*-values of Fisher’s exact tests.

Parameter	Suicide			Homicide			Odds Ratio	Significance
	*n*	f (%)	m (%)	*n*	f (%)	m (%)	(OR)	*p*-Value
Sex	15	20.0%	80%	26	46.2%	53.8%	0.3	0.177
Presence BL	6	16.7%	83.3%	18	61.1%	38.9%	3.27	0.102

## Data Availability

Not applicable.
